# Molecular Cloning and Gene Expression Analysis of Ercc6l in Sika Deer (*Cervus nippon hortulorum*)

**DOI:** 10.1371/journal.pone.0020929

**Published:** 2011-06-14

**Authors:** Yupeng Yin, Lina Tang, Jiabao Zhang, Bo Tang, Ziyi Li

**Affiliations:** 1 Jilin Provincial Key Laboratory of Animal Embryo Engineering, The Center for Animal Embryo Engineering of Jilin Province, College of Animal Science and Veterinary Medicine, Jilin University, Changchun, Jilin, China; 2 Ministry of Education Key Laboratory of Enzyme Engineering, College of Life Sciences, Jilin University, Changchun, Jilin, China; Instituto Nacional de Câncer, Brazil

## Abstract

**Background:**

One important protein family that functions in nucleotide excision repair (NER) factors is the SNF2 family. A newly identified mouse ERCC6-like gene, Ercc6l (excision repair cross-complementing rodent repair deficiency, complementation group 6-like), has been shown to be another developmentally related member of the SNF2 family.

**Methodology/Principal Findings:**

In this study, Sika deer Ercc6l cDNA was first cloned and then sequenced. The full-length cDNA of the Sika deer Ercc6l gene is 4197 bp and contains a 3732 bp open reading frame that encodes a putative protein of 1243 amino acids. The similarity of Sika deer Ercc6l to Bos taurus Ercc6l is 94.05% at the amino acid sequence level. The similarity, however, is reduced to 68.42–82.21% when compared to Ercc6l orthologs in other mammals and to less than 50% compared to orthologs in Gallus gallus and Xenopus. Additionally, the expression of Ercc6l mRNA was investigated in the organs of fetal and adult Sika deer (FSD and ASD, respectively) by quantitative RT-PCR. The common expression level of Ercc6l mRNA in the heart, liver, spleen, lung, kidney, and stomach from six different developmental stages of 18 Sika deer were examined, though the expression levels in each organ varied among individual Sika deer. During development, there was a slight trend toward decreased Ercc61 mRNA expression. The highest Ercc6l expression levels were seen at 3 months old in every organ and showed the highest level of detection in the spleen of FSD. The lowest Ercc6l expression levels were seen at 3 years old.

**Conclusions/Significance:**

We are the first to successfully clone Sika deer Ercc6l mRNA. Ercc6l transcript is present in almost every organ. During Sika deer development, there is a slight trend toward decreased Ercc61 mRNA expression. It is possible that Ercc6l has other roles in embryonic development and in maintaining the growth of animals.

## Introduction

The SNF/SWI protein family is known to play key roles in the regulation of eukaryotic gene expression. A considerable amount of genetic evidence in yeast has implicated the SNF2 protein in the antagonization of chromatin-mediated repression of transcription and in spermatogenesis, embryogenesis, and cell growth and division [1]–[6]. Several proteins with significant amino acid sequence similarity to SNF2 have been grouped into a family that contains diverse proteins with various intracellular functions. These include transcriptional regulation (SNF2, BRM and MOT1), the maintenance of chromosome stability during mitosis and various aspects of DNA damage processing, including post-replication daughter strand gap repair (RAD5), nucleotide excision repair (RAD16, RAD26 and ERCC6) and the recombinational repair pathway (RAD54) [7]–[10]. Ercc6 is one of the NER enzymes, which play pivotal roles in the maintenance of chromosome integrity and in the elimination of pre-mutagenic DNA lesions [11]–[14]. NER enzymes are critical for the removal of bulky DNA adducts caused by environmental carcinogens such as smoking. Of these enzymes, Cockayne syndrome complementation group B (CSB), which is coded by ERCC6, recruits NER repair factors to the DNA damage site and plays an important role in the repair process. Genetic variants of ERCC6 may alter the regulation of DNA repair, and are therefore hypothesized to be associated with an altered risk of smoking-related lung cancer [15]–[18].

A newly identified mouse ERCC6-like gene, Ercc6l (accession number AY172688), has been shown to be another developmentally related member of the SNF2 family [10]. Ercc6l may play a role in the early development of the mouse embryo, particularly in the central nervous system and heart; it may also play a role in the teratogenic action of alcohol. Alteration of the homologous human Ercc6l gene may be a candidate biomarker for FAS [19]. Here, we characterize a novel Ercc6 ortholog in Sika deer, designated as Ercc6l (excision repair cross-complementing rodent repair deficiency, complementation group 6-like). The GenBank accession number for Ercc6l is HQ529500. This gene was first identified in the heart of Sika deer and then verified by RT-PCR and RACE. Expression pattern analysis suggests that Ercc6l might be implicated in embryonic development and tumorigenesis.

## Results

### cDNA Cloning and Sequence Analysis of Sika Deer Ercc6l

**Figure 1:**
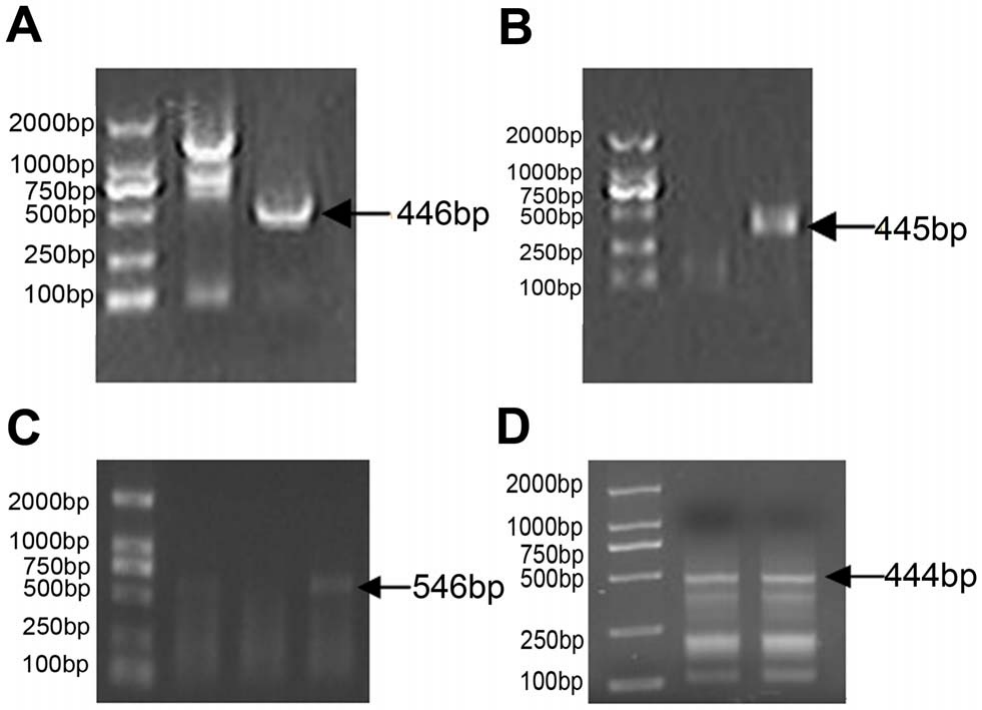
The cDNA sequences of Sika deer were obtained from heart mRNA using 5′ RACE and 3′ RACE. (A) Products obtained during the first 3′ RACE were 446 bp in length. (B) Products obtained during the second 3′ RACE were 445 bp in length. (C) Products obtained during a third 3′ RACE were 546 bp in length. (D) 5′ RACE products were 444 bp in length.

**Figure 2:**
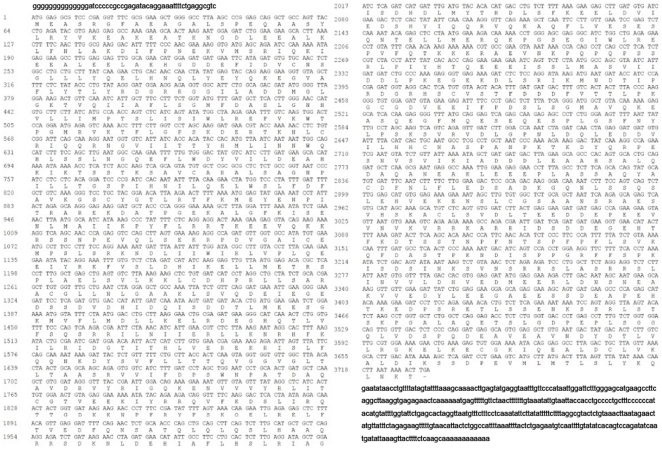
Nucleotide and deduced amino acid sequences of Ercc6l. The ORF is presented in upper case, whereas the 5′-UTR and 3′-UTR sequences are presented in lower case. The putative amino acid sequence (total 1243 a.a.) is shown under the triplet codon. The stop codon is marked by an transverse line.

The newly discovered Ercc6l cDNA (accession no. HQ529500) was cloned by RT-PCR, resulting in a 3063 bp product, and RACE, resulting in a 1134 bp product (Fig. 1). The full length of the Ercc6l cDNA was 4,197 nucleotides, consisting of a 51 nucleotide 5′UTR and 414 nucleotide 3′ UTR with a 12 nucleotide poly(A) tail. The Ercc6l cDNA also contained a 3732 nucleotide open reading frame (ORF) encoding a putative polypeptide of 1243 amino acids with a predicted molecular weight of 140.33 kDa and a theoretical isoelectric point of 5.06. The predicted Ercc6l terminus had features consistent with those of a signal peptide, as defined by SignalP analysis (http://www.cbs.dtu.dk/services/SignalP/). The predicted polypeptide consists of a 1225 amino acid mature peptide and an 18 amino acid signal peptide (Fig. 2) with a predicted cleavage site between amino acid positions 18 and 19. Similar to Ercc6l in other species, Sika deer Ercc6l also contained a large N-terminal extracellular domain; five transmembrane domains (TMD) containing different amino acids (at positions 114–171,190–218,240–286,308–326,528–556) were predicted.

### Homology Analysis of Sika deer Ercc6l

**Figure 3:**
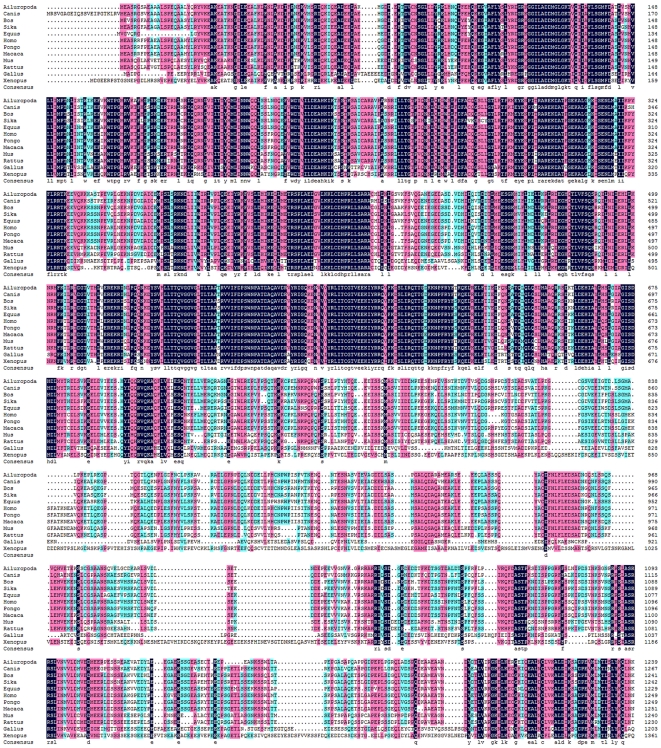
ClustalX alignment of the Ercc6l protein sequence with other members of SNF2 family as reported in other mammals. The conserved amino acid residues in these sequences are shaded to show homology.

Alignment of Sika deer Ercc6l with the amino acid sequences of other organisms showed that they shared similarities and had conserved amino acid sequences (Table 1). The amino acid sequence of Sika deer Ercc6l showed 94.05% similarity with Bos taurus Ercc6l. This similarity, however, was reduced to 68.42–82.21% when compared to Ercc6l orthologs in other mammals. When compared to Ercc61 orthologs in Gallus gallus and Xenopus, Sika deer Ercc61 showed less than 50% sequence homology. Multiple sequence alignment (Fig. 3) revealed that the sequence was highly conserved. SMART program analysis revealed that the Ercc6l-encoded polypeptide contained both a conserved domain and amino acid residues that are critical for its fundamental structure and function.

**Table 1:** Ercc6l identities between Sika deer and 12 other organisms.

Matched species	Accession No.	%Identity
Bos taurus (cattle)	NM_001102530	94.05
Equus caballus (horse)	XM_001488314	82.21
Pongo abelii (pongo)	XM_002831796	80.91
Human Homo sapiens (human)	NM_017669	80.59
Monke Macaca mulatta(monkey)	XM_001092609	80.57
Panda Ailuropoda melanoleuca (panda)	XP_002925544	80.29
Dog Canis lupus familiaris (dog)	XM_549075	78.76
Marmoset Callithrix jacchus (marmoset)	XM_002762987	77.31
Mouse Mus musculus (mouse)	NM_146235	69.13
Rat Rattus norvegicus (rat)	XM_228546	68.42
Fowl Gallus gallus (fowl)	XM_420137	48.98
Xenopus tropicalis (xenopus)	XP_002936822	43.77

### Phylogenetic Analysis of Sika deer Ercc6l

**Figure 4:**
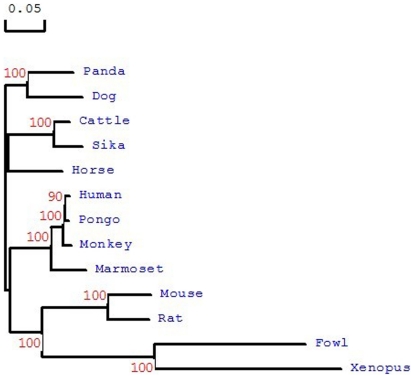
A phylogenic tree showing the similarity between Sika deer Ercc6l protein amino acid sequences and those of cattle, panda, dog, horse, fowl, human, monkey, mouse, pongo, rat, xenopus and marmoset. The number at each node indicates the percentage of bootstrapping after 1000 replications. The bar at the bottom indicates 5% amino acid divergence in the sequences. These proteins are highly conserved throughout evolution; high similarity matches exist between Ercc6l sequences across species.

To evaluate the evolutionary molecular relationships between Sika deer Ercc6l and Ercc61 orthologs of other species, a phylogenetic tree was constructed based on amino acid sequences using the neighbor-joining method (Fig. 4). There were two main branches with a strong bootstrap in the phylogenetic tree: the mammalian family members were clustered into a branch that was separate from the branch containing Gallus gallus and Xenopus. The results of the phylogenetic analysis were in accordance with the concept of traditional taxonomy.

### Tissue distribution of Sika deer Ercc6l mRNA

**Figure 5:**
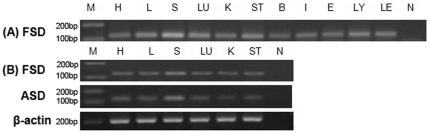
Organs distribution of fetal Sika deer (labeled FSD) and adult Sika deer Ercc6l (labeled ASD). mRNAs in the Sika deer were detected by RT-PCR. (a) After 25 cycles, FSD was detected in the heart (H), liver (L), spleen (S), lung (LU), kidney (K), stomach (ST), brain (B), intestines (I), ear (E), lymph (LY), leg (LE) and negative (N); (b) FSD and ASD were detected in organs such as heart (H), liver (L), spleen (S), lung (LU), kidney (K), stomach (ST) and negative (N).

For tissue distribution analysis, the PCR cycle number was optimized at 25 cycles, which was found to be in the mid phase of PCR amplification. With this number of cycles, Ercc6l transcript could be detected in all examined tissues and was found in ASD at the highest level in the spleen (Fig. 5A,B) and the lowest level in the kidney (Fig. 5B).

### Quantitative Analysis of Sika deer Ercc6l mRNA Expression

**Figure 6:**
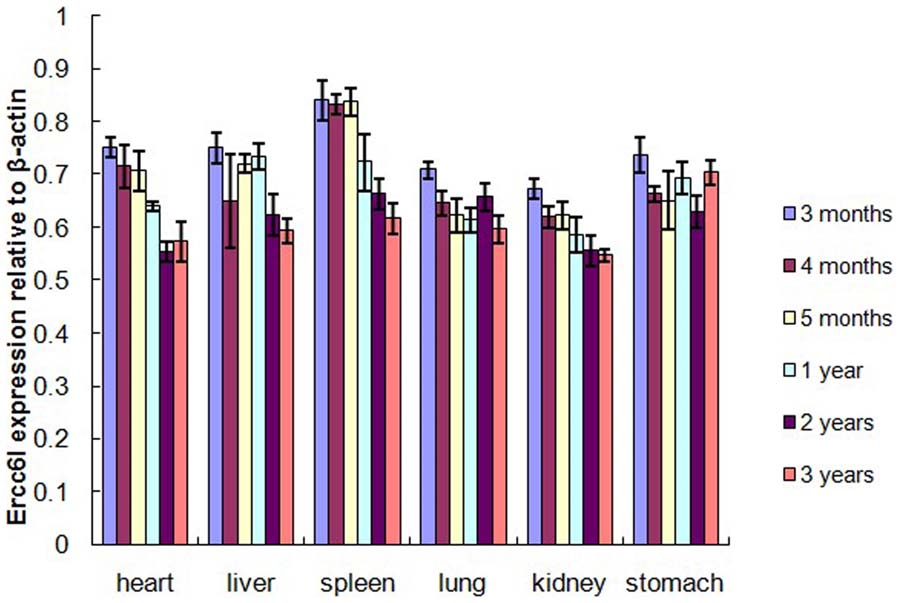
Organs distribution of the Sika deer Ercc6l transcript as measured by SYBR Green RT-PCR. Quantitative analysis was performed on Ercc6l gene expression relative to β-actin in different Organs and different developmental stages. Ercc6l gene expression was analyzed in the following Organs: heart, liver, spleen, lung, kidney and stomach. Expression data for each Organ were analyzed from three individual Sika deer. Vertical bars represent the mean±S.E.

In fetal and adult Sika deer, the mRNA expression levels of Ercc6l in six organs (heart, liver, spleen, lung, kidney and stomach) from FSD and ASD plus β-actin as an internal control were measured by real-time RT-PCR (Fig. 6). Ercc6l mRNA expression levels at different developmental stages were relatively abundant in every tissue sample. The mRNA expression levels in FSD were slightly higher compared to ASD. With the development of Sika deer, there was a slight trend toward slightly lower mRNA expression levels. The highest Ercc6l expression levels were seen at three months in the spleen of FSD. The expression levels of Ercc6l in the spleen were significantly higher in FSD than in ASD (P<0.01), and a similar pattern of Ercc6l expression was seen in the heart (P<0.05). However, Ercc6l expression in the liver, lung, kidney and stomach was not significantly higher in FSD than in ASD (P>0.05). Finally, the lowest Ercc6l expression levels in all organ samples were seen at three years old.

## Discussion

The SNF2/SWI2 ATPase/helicase family consists of proteins from a variety of species and serves a number of functions, including transcriptional regulation, maintenance of chromosome stability during mitosis, and various types of nucleotide excision repair (NER). Several proteins with unknown functions are also included in this family. Among these functions, the most important is the NER mechanism, which plays a pivotal role in the maintenance of chromosome integrity and in the elimination of premutagenic lesions from DNA [11]–[14].

The number of genes belonging to this family is rapidly expanding, making it easier to analyze the common biological functions of the family members. Due to differences between species, the gene functions are not necessarily conserved. Therefore, it is best to study the expression of the same gene across species to see if there are significant differences. To date, many genes have been identified and functionally characterized in mammals, however relatively few molecular or functional studies on Sika deer genes have been conducted.

In the present study, Sika deer Ercc6l was cloned for the first time. The full-length Ercc6l cDNA (accession no. HQ529500), which is located on chromosome X and made up of two exons, was shown to encode a predicted polypeptide of 1243 amino acids, with a theoretical mass of 140.33 kDa and an isoelectric point of 5.06. Similar to Ercc6l in other species, Sika deer Ercc6l also contained a large N-terminal extracellular domain and five transmembrane domains (TMD) that contained different predicted amino acids. There were also other ERCC6-like features including ATP-dependent helicase, DNA excision repair protein, Plk1-interacting checkpoint helicase, SNF2/RAD54 family protein and excision repair cross-complementing rodent repair deficiency complementation groups.

The Sika deer is a single-birth artiodactyla and a ruminant animal like Bos taurus. The amino acid sequence of Sika deer Ercc6l showed 94.05% similarity to Bos taurus Ercc6l. This similarity, however, was reduced to 68.42–82.21% when compared to Ercc6l orthologs in other mammals, and to less than 50% compared to orthologs in Gallus gallus and Xenopus. These data show the relative genetic relationship of Sika deer with other species, suggesting that Sika deer and Bos taurus are the closest genetically, relative to other mammals, and Gallus gallus and Xenopus are the most distant.

We then detected the distribution of Sika deer Ercc6l mRNA. In FSD, Ercc61 was detected in the heart (H), liver (L), spleen (S), lung (LU), kidney (K), stomach (ST), brain (B), intestines (I), ear (E), lymph (LY) and leg (LE). In ASD, Ercc61 was detected in the heart (H), liver (L), spleen (S), lung (LU), kidney (K) and stomach (ST). Ercc6l transcript was predominantly expressed in heart (H), liver (L), spleen (S), lung (LU), kidney (K), stomach (ST), brain (B), intestines (I), ear (E), and lymph (LY). In Sika deer, these organs are all important for immune defense against pathogens (Fig. 5). The strongest signal was seen in the spleen. These findings were consistent with our fluorescence quantitative results. In a previous study, however, the analysis of the expression pattern of mouse Ercc61 based on EST or RT-PCR suggested that relative expression levels varied with organs and were down regulated through the course of development [19].

We detected the mRNA expression level of Ercc6l in six organs (heart, liver, spleen, lung, kidney and stomach) through six different developmental stages from FSD and ASD, and β-actin was measured by real-time RT-PCR as an internal control. The Ercc6l mRNA expression levels at six different developmental stages were relatively abundant in every organ tested. However, Ercc6l transcripts were detected at a lower level in the kidneys of 3-year-old ASD. This distinct expression of Ercc6l in six organs may correspond to different subsets and maturation statuses of these organs.

In general, expression levels of Ercc6l were not significantly different in the six organs across the different developmental stages between FSD and ASD. The Ercc6l expression levels in FSD were slightly higher than in ASD. During Sika deer development, there was a slight trend toward slightly lower Ercc6l mRNA expression levels (Fig. 6). The highest Ercc6l expression levels in every organ sample were seen at three months old. The spleen is well known as an important lymphoid organ for immune defense against pathogens. The expression levels of Ercc6l in the spleen were significantly higher in FSD than that in ASD (P<0.01), suggesting a role for Ercc6l in the spleen at developmental stages; the same is true for Ercc6l expression in the heart (P<0.05). However, Ercc6l expression in the liver, lung, kidney and stomach at different developmental stages was not significantly different in FSD compared to ASD (P>0.05); Ercc6l expression in lung and stomach were almost equal between FSD and ASD. These findings suggest that Ercc6l expression remains steady in the lung and stomach across different developmental stages. Finally, the lowest Ercc6l expression levels were seen at three years old in every organ. These findings suggest that Ercc6l is a putative developmentally related molecule in Sika deer.

In conclusion, we are the first to have successfully cloned Sika deer Ercc6l mRNA. Ercc6l transcript was predominantly expressed in almost all organs. During Sika deer development, there is a slight trend toward a decrease in the mRNA expression level of Ercc6l. It is possible that Ercc6l has different roles in embryo development and in maintaining animal growth.

## Materials And Methods

### Ethics Statement

All animal studies were conducted according to the experimental practices and standards approved by the Animal Welfare and Research Ethics Committee at Jilin University (Approval ID: 20091008-1).

### Bioinformatics

Alignment of cDNA sequences and percentage similarity analyses were performed using the CLUSTALX multiple alignment tool (http://www.ebi.ac.uk/clustalw) [20]. BLAST analysis (National Center for Biotechnology Information [NCBI]; http://www.ncbi.nlm.nih.gov/) was used to search for Ercc6l mRNA and EST sequences.

### Animals and tissue collection

Fetal Sika deer were obtained from normal oocyte fertilization at a local farm. The day of insemination was considered as day 0, and the pregnant Sika deer were about euthanized on months 3, 4 or 5 of gestation. All Sika deer were euthanized by a jugular vein bleed. All collected organs were thoroughly rinsed in phosphate buffered saline (PBS) to remove hemocytes. The organ samples (0.5×1.0×1.0 cm in size), including those from the heart, liver, spleen, lung, kidneys and stomach, were collected immediately from fetuses, frozen in liquid nitrogen and stored at −80°C until use. Adult Sika deer were euthanized on year 1, 2 or 3, and different organs including the heart, liver, spleen, lung, kidneys, and stomach were collected, frozen in liquid nitrogen, and stored at −80°C until use. There were three replicates for each age and three replicates for each tissue. A total of 18 organ samples were used in this study. All procedures were approved by Jilin University Animal Care Committee.

### Total RNA isolation and cDNA Synthesis

Total RNA was extracted from homogenized organ samples obtained from FSD and ASD using the Trizol Reagent (Invitrogen, USA) according the manufacturer and dissolved in diethyl pyrocarbonate (DEPC)-treated H_2_O. The presence of RNA was confirmed by 1% formaldehyde agarose gel electrophoresis and quantified using a spectrophotometer (Thermo, USA). Total RNA was cleaned of contaminating DNA using the RNeasy Mini Kit (Invitrogen, USA), and mRNA was purified using the mRNA purification kit (Amersham Pharmacia, USA) according to the manufacturer's instructions. Total RNA (1 µg) from each sample was reverse-transcribed using the BioRT cDNA First Synthesis kit (BioRT). Aliquots of cDNA made from tissue RNA were used as the templates for real time PCR and rapid amplification of cDNA ends (RACE) reactions using primers for Ercc6l.

### Cloning of Ercc6l open reading frames

In Sika deer, the Ercc6l gene is uncharacterized. We used BLAST analysis (National Center for Biotechnology Information [NCBI]; http://www.ncbi.nlm.nih.gov/) to search for Ercc6l mRNA and EST sequences. Because cattle and Sika deer show close genetic homology, two pairs of primers (Table 2: E-1, E-2) were designed according to the sequence of cattle Ercc6l mRNA. All incubations were performed with a thermal cycler in 0.2 ml tubes. A 25 µl PCR reaction mixture containing 9 µl dH_2_O, 13 µl 2× Master Mix (TIANGEN), 1 µl each of the gene specific primers E-1 and E-2 (10 µM) and 1 µl heart cDNA. PCR amplification was performed using the cDNA template from the heart of an FSD (1 cycle of 94°C for 3 min; 35 cycles of denaturation at 94°C for 30 sec, annealing at 57°C and 60°C for 30 sec and extension at 72°C for 1 min; followed by 1 cycle of 72°C for 10 min). The PCR products were analyzed on 1.5% agarose gels, with product sizes of 1624 bp and 1652 bp representing the two parts of Ercc6l mRNA. The RT-PCR product mixture was ligated into the pMD™18-T Vector (TaKaRa, China) at 12°C overnight. The ligation solution was then transformed into competent E. coli. DH5α cells were cultured on LB plates with 100 mg/ml ampicillin. Positive colonies were screened by PCR and subsequently extracted for sequence confirmation.

**Table 2:** Ercc6l Primers and PCR conditions.

Primers Names	Primer (5′ to 3′)	Annealing temperature(°C)×cycle number	Product size (bp)
E-1	Forward: GCTGCCAGTTACCTGAGAT	57×30	1624
	Reverse: GTTAAGCCAACACCACCTAC	57×30	
E-2	Forward:CTCTGTTTTTCTGCTTACCACTC	60×30	1562
	Reverse: TGGGTTTGTGCTTGAAGTATC	60×30	
E-3′RACE1	Outer1: GGAACCTTTAGCCTCTTCAGCA	60×30	446
	Inner1: GCAAAGCATGTCTCAGTGTG	58×30	
E-3′RACE2	Outer2: ACCCATTCAACACATCTCCCTTCC	61×30	445
	Inner2:CAATGAAGCAAAGGTTGTTGAAGA	57×30	
E-3′RACE3	Outer3: TGAGGCAGTGGAGGCTGTGAAT	61×30	546
	Inter3: AAAGAACTGAAAGAGTGTGG	54×30	
E-5′RACE	GSP1: TGACTTGGTAGATGAGGT	42×30	444
	GSP2:CTAGGACCAAGAAAGGTTTTGACTC	60×30	
	GSPs: TTGAACAGTCTTTCCTAACCCCAT	60×30	
E-SYBR	Forward: TGAATGATACCATCCCACG	56.9×40	132
	Reverse: CAGCCATCCCTGATAAAGA	56.9×40	
β-actin	Forward: CCCAGATCATGTTCGAGACT	55.7×40	256
	Reverse: TCGGCTGTGGTGGTAAAG	55.7×40	

### 5′ and 3′ Rapid amplification of cDNA ends (RACE)

Full-length cDNA sequences were obtained from heart mRNA using the 5′ RACE System for Rapid Amplification of cDNA Ends Kit (Version 2.0, Invitrogen) and 3′-Full RACE (TaKaRa Kit) according to the manufacturers' protocols. Briefly, 3′ RACE took advantage of the natural poly(A) tail in mRNA as a generic priming site for PCR amplification. In this procedure, mRNAs were converted into cDNA using reverse transcriptase (RT) and an oligo-dT adapter primer. Specific cDNA was then directly amplified by PCR using a gene-specific primer that anneals to a region of known exon sequences and an adapter primer that targeted the poly(A) tail region. This permits the capture of unknown 3′-mRNA sequences that lie between the exon and the poly(A) tail. 5′ RACE, or “anchored” PCR, is a technique that facilitates the isolation and characterization of 5′ ends from low-copy messages [21]. Although the precise protocol varies between users, the general strategy remains consistent. First strand cDNA synthesis was primed using a gene-specific antisense oligonucleotide (GSP1). This permits cDNA synthesis from a specific mRNA or related families of mRNAs and maximizes the potential for complete extension to the 5′-end of the message. Following cDNA synthesis, the first strand product was purified from unincorporated dNTPs and GSP1. TdT (Terminal deoxynucleotidyl transferase) was used to add homopolymeric tails to the 3′ ends of the cDNA. In the original protocol, tailed cDNA was then amplified by PCR using a mixture of three primers: a nested gene-specific primer (GSP2), which anneals 3′ to GSP1 and a combination of a complementary homopolymer-containing anchor primer and corresponding adapter primer, which permitted amplification from the homopolymeric tail. This allowed for the amplification of unknown sequences between the GSP2 primer binding site and the 5′-end of the mRNA.

The gene-specific primers used (Table 2: E-3′RACE1, E-3′RACE2, E-3′RACE3, E-5′RACE) were based on the available mRNA sequence for Sika deer Ercc6l. The RT-PCR product mixture was ligated into the pMDTM18-T Vector (TaKaRa, China) at 12°C overnight. The ligation solution was then transformed into the competent E. coli. DH5α cells were cultured on LB plates with 100 mg/ml ampicillin. The positive colonies were screened by PCR and then extracted from their plasmids and confirmed by sequencing

### Sequence Analysis, Multiple Sequences Alignment, and Phylogenetic Analysis

Sequence similarity analyses were performed using the BLAST program at the National Center for Biotechnology Information (http://www.ncbi.nlm.nih.gov/blast). The open reading frame (ORF) for the Ercc6l cDNA was determined using ORF Finder (www.ncbi.nlm.nih.gov/gorf/) and translated into the corresponding amino acid sequence. The predicted signal peptide sequence was identified using the Signal 3.0 Server (www.cbs.dtu.dk/services/SignalP). Protein motif features were predicted using the Simple Modular Architecture Research Tool (SMART) (http://www.smart.emblheidelberg.de). Sequence alignments were determined using the ClustalX software package. The sequences derived from the NCBI database are represented using their official names. The protein sequences were aligned using the Clustal program [22]. Phylogenetic analyses and statistical neighbor-joining bootstrap tests of the phylogenies were carried out using MEGA version 3.0 [23]. Theoretical pI values and predicted molecular masses were calculated using Prot-Param tools (http://kr.expasy.org/tools/protparam.html).

### Quantitative real-time PCR (qRT-PCR) analysis of Ercc6l expression in six organs at six different developmental stages

To determine the expression level of Ercc6l in different organs (heart, liver, spleen lung, kidneys and stomach) of healthy FSD (in the rapidly developing stage) and in the same organs of healthy ASD, total RNA extracted from each organ was subjected to qRT-PCR analysis. All organs were thoroughly rinsed with phosphate buffered saline (PBS) buffer to remove hemocytes. Single-strand cDNA was synthesized from about 5 µg of total RNA by using the Revertaid™ First Strand cDNA Synthesis kit (Fermentas) and diluted 10 times. Filtered distilled water was used as the negative control. Template without reverse transcriptase was used to check for contaminating genomic DNA. A pair of gene specific primers (Table 2: E-SYBR) were designed to amplify an Ercc61 transcript product of 132 bp, and the PCR product was sequenced to verify the specificity of the PCR primers. Primers for β-actin (Table 2) were used to amplify a 256 bp fragment of the beta-actin gene as a reference. A SYBR Green RT-PCR assay was conducted to determine the Ercc6l mRNA expression. The PCR temperature profile and reaction conditions were specified by the manufacturer of the SYBR Premix Ex Taq™ (TaKaRa, China) on an ABI step two real-time PCR system (Applied Biosystems 7500, USA). The reaction was performed at 95°C for 30 s followed by 40 cycles of 95°C for 5 s, 55.7°C or 56.9°C (for β-actin and E-SYBR, respectively) for 34 s to obtain the dissociation curves. The standard curves tested derived from a series of diluted cDNA (10-fold serial dilution with ten levels: 10^10^×, 10^9^×, 10^8^×, 10^7^×, 10^6^×, 10^5^×, 10^4^×, 10^3^×, 10^2^×, 10×, for β-actin and E-SYBR). The R^2^ values of the standard curves were 0.999175 and 0.999712, and the dissociation curves showed a single peak. The relative ratios of E-SYBR to β-actin mRNA were calculated using the standard curves to confirm the reliability of the real-time PCR data.

### Statistical analysis

Data obtained from the real-time PCR analysis were subjected to ANOVA, and a t-test was used to determine the difference in mean values using SPSS software. The P value for significance was set at P≤0.05.
